# Subverting lysosomal function in *Trypanosoma brucei*

**DOI:** 10.15698/mic2015.08.222

**Published:** 2015-08-03

**Authors:** Sam Alsford

**Affiliations:** 1 London School of Hygiene & Tropical Medicine, Keppel Street, London WC1E 7HT, UK.

**Keywords:** Trypanosoma brucei, lysosome, LeuLeu-OMe

## Abstract

In this issue of *Microbial Cell*, Koh and colleagues present data highlighting the utility of the lysosomotropic compound L-leucyl-L-leucyl methyl ester (LeuLeu-OMe) as an anti-*Trypanosoma brucei* agent, adding to the range of compounds that either directly target lysosomal enzymes or that can be used to subvert the function of the lysosome for parasite destruction.

*Trypanosoma brucei* causes devastating diseases in humans and cattle 1. Human African trypanosomiasis (HAT), caused by *T. b. gambiense* and *T. b. rhodesiense*, and the livestock disease Nagana, caused by *T. brucei* and related parasites, are endemic to sub-Saharan Africa, with approximately 60 million people living in at risk areas 2. Due to sustained control efforts over the last two decades, HAT cases have dropped from an estimated peak of 300-500 thousand per year in the 1990s, to less than 10,000 reported cases per year since 2009 3. Given this success, the World Health Organisation has stated that HAT should be eliminated as a public health problem by 2020 4. However, *T. brucei* and the related zoonotic African trypanosomes, *T. congolense* and *T. vivax*, still present a significant constraint on agricultural development in the region. Meeting the HAT elimination goal in an environment of widespread zoonotic trypanosomiasis will likely require the development of new chemotherapeutic interventions for both HAT and Nagana. The current arsenal of anti-HAT drugs is limited by disease stage and sub-species specificity, as well as toxicity and the need for complex administration protocols 5. In addition, resistance to the anti-HAT drugs, melarsoprol and pentamidine, has been extensively characterised in clinical isolates 6, and treatment failures with the anti-animal trypanosomiasis drugs, diminazene aceturate and isometamidium chloride are also seen 7.

A small number of new compounds are currently under clinical development for the treatment of HAT, including orally available oxaboroles 8 and fexinidazole 9. Work is also ongoing to identify specific trypanosomal drug targets, inspired by the array of unique features present in this evolutionarily divergent eukaryote, which represent attractive targets for chemotherapeutic intervention 10. Given this, it is perhaps surprising that the lysosome, an organelle found in all eukaryotes, has emerged as a promising drug target. Cathepsin-L, an essential *T. brucei* lysosomal cysteine protease 11 and its homologue in the related trypanosomatid *T. cruzi*, the causative agent of Chagas disease, are the subject of intense research 12, with the aim of developing selective inhibitors. Several other anti-parasitic agents either depend on proper lysosomal function for their efficacy, such as the anti-HAT drug suramin 13, or impact its function directly, such as the anti-malarial chloroquine 14. In this edition of *Microbial Cell*, Koh and colleagues highlight the anti-trypanosomal potential of other lysosomotropic compounds, demonstrating the selectivity of LeuLeu-OMe for bloodstream form *T. brucei* and its likely mode of killing 15. LeuLeu-OMe has previously been shown to cause lysosomal membrane permeabilisation (LMP) in *Plasmodium falciparum*, though only at millimolar concentrations 14. With an EC_50_ of 16 µM, its potency against *T. brucei* is considerably higher (Koh *et al*., 2015), although this will need to be further improved, if LeuLeu-OMe and related compounds are to have a future role in HAT chemotherapy.

LeuLeu-OMe exhibits some toxicity against mammalian cells, with selectivity for cytotoxic lymphocytes 16, but even these cells are less sensitive to this compound than *T. brucei* (Koh *et al.*, 2015). Treatment of HeLa cells with >300 µM LeuLeu-OMe results in LMP and the release of cathepsins into the cytosol, which cleave the key apoptosis regulator, Bid, leading to caspase activation and apoptosis 17. The phenotypes seen in *P. falciparum* following LMP are also reminiscent of apoptosis, and include mitochondrial membrane depolarisation and DNA fragmentation 14. Apoptosis-like features have also been reported in *T. brucei* and other protozoa 18, however the absence of the key regulators and the caspase executioners, and the lack of convincing evidence for regulation of the process have led others to conclude that the observed cell death should be described as incidental or necrotic 19. Indeed, although cathepsin release following LMP can lead to apoptosis in metazoa 17, the data presented by Koh and colleagues indicate that LeuLeu-OMe mediated LMP in *T. brucei* causes necrotic cell death, without any discernible markers of apoptosis 15. One or both of the parasite’s cathepsins (B and L) appears to be at least partly responsible for the observed necrosis, as incubation of LeuLeu-OMe treated cells with a broad range cathepsin inhibitor significantly reduces cell death 15.

The lysosome has long been recognised as a viable target for chemotherapeutic intervention that subverts its function for parasite destruction. Indeed, humans are resistant to infection by the non-human infectious African trypanosomes, such as *T. congolense* and *T. b. brucei*, due to serum factors that target the parasite’s lysosome. Human serum contains two trypanolytic complexes, which rapidly lyse non-human infectious African trypanosomes: trypanolytic factor (TLF) 1 is a component of high density lipoprotein, while TLF2 is composed primarily of IgM 20. The lytic component of both complexes is apolipoprotein-L1, which forms pores in the lysosomal membrane following a conformational change at low pH, allowing the uncontrolled entry of chloride ions into the lysosome and its subsequent osmotic swelling leading to parasite lysis 21. The lysosome and the endocytic system that feeds into it are also attractive drug targets by virtue of their fundamental role in surface coat recycling and antigenic variation, the process whereby *T. brucei* changes its variant surface glycoprotein coat, enabling immune evasion 22 and the rapid removal of bound antibody 23. It may be these features, the very high endocytic rate and a necessarily highly active lysosome, that render bloodstream form *T. brucei* more sensitive than human cells to LeuLeu-OMe.

The importance of the endosomal-lysosomal system to *T. brucei* survival in the mammalian host, both as a route for nutrient uptake and for surface coat maintenance, underpins its successful targeting by both the innate immune system and chemotherapy 24. Any new compounds that target this area are an attractive proposition. Whether, LeuLeu-OMe represents a significant step forward will depend on the ease with which more potent and highly selective variants can be developed.
